# Ionically Conductive Tunnels in *h*‐WO_3_ Enable High‐Rate NH_4_^+^ Storage

**DOI:** 10.1002/advs.202105158

**Published:** 2022-02-02

**Authors:** Yi‐Zhou Zhang, Jin Liang, Zihao Huang, Qian Wang, Guoyin Zhu, Shengyang Dong, Hanfeng Liang, Xiaochen Dong

**Affiliations:** ^1^ School of Chemistry and Materials Science Institute of Advanced Materials and Flexible Electronics (IAMFE) Nanjing University of Information Science and Technology Nanjing 210044 China; ^2^ Key Laboratory of Flexible Electronics (KLOFE) & Institute of Advanced Materials (IAM) Nanjing Tech University (NanjingTech) Nanjing 211816 China; ^3^ State Key Laboratory of Physical Chemistry of Solid Surfaces College of Chemistry and Chemical Engineering Xiamen University Xiamen 361005 China

**Keywords:** energy storage, ionic tunnels, NH_4_^+^, WO_3_

## Abstract

Compared to the commonly applied metallic ion charge carriers (e.g., Li^+^ and Na^+^), batteries using nonmetallic charge carriers (e.g., H^+^ and NH_4_^+^) generally have much faster kinetics and high‐rate capability thanks to the small hydrated ionic sizes and nondiffusion control topochemistry. However, the hosts for nonmetallic charge carriers are still limited. In this work, it is suggested that mixed ionic–electronic conductors can serve as a promising host for NH_4_^+^ storage. Using hexagonal tungsten oxide (*h*‐WO_3_) as an example, it is shown that the existence of ionic conductive tunnels greatly promotes the high‐rate NH_4_^+^ storage. Specifically, a much higher capacity of 82 mAh g^–1^ at 1 A g^–1^ is achieved on *h*‐WO_3_, in sharp contrast to 14 mAh g^–1^ of monoclinic tungsten oxide (*m*‐WO_3_). In addition, unlike layered materials, the insertion and desertion of NH_4_^+^ ions are confined within the tunnels of the *h*‐WO_3_, which minimizes the damage to the crystal structure. This leads to outstanding stability of up to 200 000 cycles with 68% capacity retention at a high current of 20 A g^–1^.

## Graphical abstract

Hexagonal tungsten oxide (*h*‐WO_3_) with mixed ionically and electronically conductive tunnels can serve as a promising host for high‐rate NH_4_^+^ storage. Specifically, a much higher capacity of 82 mAh g^–1^ at 1 A g^–1^ is achieved on *h*‐WO_3_, in sharp contrast to 14 mAh g^–1^ of its polymorph monoclinic tungsten oxide (*m*‐WO_3_).

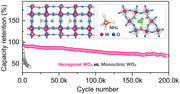

## 1 Introduction

The everlasting energy demands have driven the exponential growth of the battery market, which is currently dominated by Li‐ion batteries (LIBs).^[^ ^1^ ^]^ Other metal‐ion batteries (e.g., Na^+^, K^+^, Mg^2+^, and Zn^2+^) as alternatives to LIBs are also under rapid development aiming to reduce the cost and improve safety.^[^ ^2^, ^3^, ^4^, ^5^ ^]^ In contrast, batteries using nonmetallic ions (e.g., H^+^ and NH_4_^+^) as charge carriers have rarely been explored,^[^ ^6^, ^7^, ^8^ ^]^ although they might have ultrafast kinetics thanks to the small hydrated ionic sizes and nondiffusion control topochemistry. In addition, the use of aqueous electrolytes makes these batteries much safer and cheaper. Proton batteries often use corrosive acidic electrolytes and therefore have a high requirement for materials in the device. On the other hand, batteries using NH_4_^+^ ions as charge carriers have relatively facile operating conditions. To date, only a handful of electrode materials have been investigated for NH_4_^+^ storage, such as Prussian blue analogue with an open framework,^[^ ^9^ ^]^ Ti_3_C_2_ MXene,^[^ ^10^ ^]^ and V_2_O_5_ with layered structures.^[^ ^7^ ^]^ It is therefore of great interest to explore new materials to further advance the NH_4_^+^ batteries.

**Figure 1:**
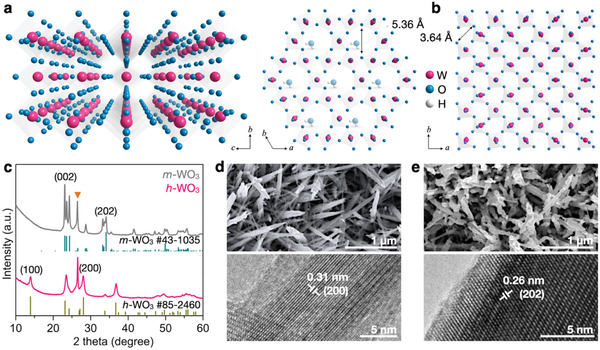
Structure characterization of WO_3_. a) Crystal structure of *h*‐WO_3_ viewed from *a* (left) and *c* (right) axes. b) Crystal structure of *m*‐WO_3_ viewed from *c* axis. c) XRD patterns of *h*‐WO_3_ and *m*‐WO_3_. The triangle marks the diffraction peak from the carbon paper substrate. d) SEM (top) and HRTEM (bottom) images of *h*‐WO_3_. e) SEM (top) and HRTEM (bottom) images of *m*‐WO_3_.

The energy storage in NH_4_^+^ batteries is achieved by the simultaneous translocation of NH_4_^+^ ions and electrons.^[^ ^8^ ^]^ In this regard, mixed ionic–electronic conductors (MIECs) ^[^ ^11^, ^12^ ^]^ could play an important role in enhancing electrochemical performance. MIECs can conduct both ions and electrons simultaneously, which is more efficient and easier than building two materials that conduct ions and electrons separately. MIECs such as LiCoO_2_ and LiMnO_2_ are commonly used as electrodes in LIBs,^[^ ^13^ ^]^ and their properties essentially determine the battery performance. These materials, however, often undergo the transition of crystalline phase upon Li^+^ insertion/desertion and thus suffer from slow electrode kinetics.^[^ ^14^ ^]^ Other MIECs include RuO_2_⋅H_2_O ^[^ ^15^ ^]^ and WO_3_,^[^ ^16^ ^]^ whereas the former is highly costly. The stoichiometric WO_3_ is a semiconductor but could become electronically conductive in its nonstoichiometric form (WO*_x_*, 2 < *x* < 3),^[^ ^17^ ^]^ which can be achieved by intercalating a guest ion (e.g., H^+^) into the WO_3_ matrix.^[^ ^18^ ^]^ The WO_3_‐based MIECs have already shown good electrochemical performance toward H^+^ and Li^+^ storage,^[^ ^14^, ^19^ ^]^ and we envision that they would be promising hosts for NH_4_^+^ storage. In this work, we show that the *h*‐WO_3_, whose ionic conduction relies on the water molecules within the tunnels,^[^ ^14^ ^]^ possesses significantly better NH_4_^+^ storage performance as compared to its polymorph *m*‐WO_3_. As shown in **Figure** **1**a, the *h*‐WO_3_ consists of six‐membered rings that are assembled by corner‐sharing WO_3_ octahedra. The stacking of these rings along with the *c*‐axis forms hexagonal tunnels. These tunnels are filled with water molecules, which serve as ion‐conducting chains and therefore promote the transport of NH_4_^+^ ions, similar to the biological channels.^[^ ^20^ ^]^ The water content is around 4.8 wt%, which is determined by thermogravimetric/differential scanning calorimeter (TG/DSC) analysis (Figure S1, Supporting Information). Whereas in *m*‐WO_3_, the arrangement of WO_6_ is different from that in *h*‐WO_3_ and forms a distorted ReO_3_‐type structure (Figure 1b).^[^ ^21^ ^]^ Note that there are no water molecules in *m*‐WO_3_ structure. Such structural difference results in distinctly different electrochemical behavior of *h*‐WO_3_ and *m*‐WO_3_, although they are chemically similar. Specifically, the *h*‐WO_3_ delivers a high NH_4_^+^ storage capacity of 82 mAh g^–1^ at 1000 mA g^–1^, whereas only 14 mAh g^–1^ is achieved on *m*‐WO_3_. In addition, the insertion and desertion of NH_4_^+^ ions are confined within the tunnels of the *h*‐WO_3_, which minimizes the damage to the crystal structure; therefore, high stability is achieved with no significant performance decay for 200 000 cycles even at a high current of 20 A g^–1^, in sharp contrast to that of *m*‐WO_3_ which suffers from fast capacitance drop within only 10 000 cycles. Our work not only provides a new host material for NH_4_^+^ ions but also suggests the important role of ionic conducting tunnels in enhancing electrochemical performance.

The *h*‐WO_3_ was synthesized by a facile hydrothermal reaction (see details in the Experimental Section). The diffraction peaks (Figure 1c) can be assigned to the hexagonal phase WO_3_ (JCPDS #85‐2460). After thermal annealing, the *h*‐WO_3_ was readily converted to *m*‐WO_3_ (JCPDS #43‐1035). The phase transformation did not result in significant morphological changes. Both the *h*‐WO_3_ (Figure 1d) and *m*‐WO_3_ (Figure 1e) show nanorod morphology whereas the latter appears to have a slight aggregation thanks to the thermal treatment. The lattice spacing of 0.31 and 0.26 nm from the HRTEM images can be well indexed to the (200) and (202) planes of *h*‐WO_3_ and *m*‐WO_3_, respectively. In addition, the *h*‐WO_3_ and *m*‐WO_3_ flexible electrodes have comparable electron conductivity: 38 and 51 mS cm^–1^, respectively. The synthesis of morphologically similar nanorods of *h*‐WO_3_ and *m*‐WO_3_ allows a direct comparison of their electrochemical performance and, therefore, a better understanding of the role of ionic tunnels.

**Figure 2:**
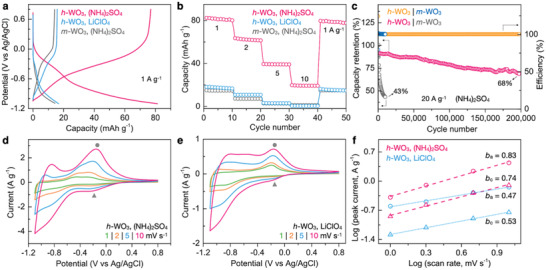
Electrochemical performance of WO_3_ electrodes. a) CD curves of *h*‐WO_3_ in 1 m (NH_4_)_2_SO_4_ or 1 m LiClO_4_ electrolytes, along with the CD curve of *m*‐WO_3_ in 1 m (NH_4_)_2_SO_4_. b) Rate performance of *h*‐WO_3_ and *m*‐WO_3_ in different electrolytes. c) Cycling performance of *h*‐WO_3_ and *m*‐WO_3_ in 1 m (NH_4_)_2_SO_4_ at 20 A g^–1^. d) CV curves of *h*‐WO_3_ in 1 m (NH_4_)_2_SO_4_ at various scan rates. e) CV curves of *h*‐WO_3_ in 1 m LiClO_4_ at various scan rates. f) Comparison of *b* values of *h*‐WO_3_ in different electrolytes.

We then tested the NH_4_^+^ storage performance of both the *h*‐WO_3_ and *m*‐WO_3_ using a three‐electrode setup, where the *h*‐WO_3_ (or *m*‐WO_3_) on carbon paper was used as the working electrode, a Ag/AgCl electrode as the reference electrode, and a graphite rod as the counter electrode. In 1 m (NH_4_)_2_SO_4_ electrolyte, the *h*‐WO_3_ delivers a high capacity of 82 mAh g^–1^ at a high current density of 1 A g^–1^, significantly larger than that of *m*‐WO_3_ (14 mAh g^–1^; **Figure** **2**a). This disparity highlights the vital role of ionic tunnels in enhancing electrochemical performance. The *h*‐WO_3_ also possesses a much higher rate capability toward the NH_4_^+^ storage in terms of capacity retention at high currents (Figure 2b). For instance, the capacity of *h*‐WO_3_ is 20 mAh g^–1^ at 10 A g^–1^, whereas it is only 1.3 mAh g^–1^ for *m*‐WO_3_. Besides the high capacity, the *h*‐WO_3_ also possesses outstanding stability with an 80% capacity retention at 100 000 cycles and 68% at 200 000 cycles under the current density of 20 A g^–1^ (Figure 2c). In sharp contrast, the *m*‐WO_3_ suffers from quick performance decay within 10 000 cycles. This result indicates the high structural stability of *h*‐WO_3_ to readily accommodate NH_4_^+^ ions within the tunnels. As shown in Figure S2 (Supporting Information), *h*‐WO_3_ has superb cycling performance under medium current density of 5 A g^–1^ without capacity fading after 5000 cycles. In addition, we also evaluated the electrochemical performance of *h*‐WO_3_ for other monovalent metal ions (e.g., Li^+^) storage and found that only a low capacity (17 mAh g^–1^) was achieved under the same current density (Figure 2a). To better understand the kinetics of these two charge carriers (metal vs nonmetal), simple CV scans at various rates were performed. Figure 2d shows the CV curves of *h*‐WO_3_ in 1 m (NH_4_)_2_SO_4_. The redox peaks at −0.15 V versus Ag/AgCl correspond to the intercalation of NH_4_^+^ ions into the tunnels. The intercalation of Li^+^ to the *h*‐WO_3_ appears at the same potential (Figure 2e, also see the charge–discharge curves in Figure S3 in the Supporting Information). However, the charge storage kinetics seems to be different. Both the faradaic (redox) reaction and double layer capacitance contribute to the overall charge storage, and the former can be further categorized into two components: the pseudocapacitive process that occurs on or near the electrode surface, and the intercalation of charge carriers into bulk materials whose process is limited by the diffusion kinetics.^[^ ^22^ ^]^ These two behaviors can be characterized by the equation: *i* = *av^b^*,^[^ ^23^ ^]^ where *i* represents the peak current, *a* is a coefficient, and *v* is the scan rate. The obtained *b* value then serves as an indicator of the charge storage type. A *b* value of 0.5 suggests a diffusion‐determined process, whereas *b* of 1 indicates a capacitive behavior. As shown in Figure 2f, the *b* values are 0.83 and 0.74 when NH_4_^+^ was used as the charge carrier, implying the charge storage is dominated by pseudocapacitive behavior, a characteristic that is believed to be favorable to the high‐rate storage. In contrast, the Li^+^ in *h*‐WO_3_ presents a mostly diffusion‐limited process. This result demonstrates that the metallic and nonmetallic charge carriers might interact with the host *h*‐WO_3_ through different bonding chemistries, and therefore resulting in distinct electrode kinetics. Further, we noticed that the *b* value of *m*‐WO_3_ with (NH_4_)_2_SO_4_ electrolyte is also smaller than 0.5 (Figure S4, Supporting Information), indicating a significantly different behavior as compared to *h*‐WO_3_. As demonstrated in Figures S5 and S6 (Supporting Information), the contribution of capacitive storage is greater for *h*‐WO_3_ than *m*‐WO_3_. This result verifies the important role of ionically conductive tunnels in enhancing the high‐rate performance of NH_4_^+^ storage by enabling the surface pseudocapacitive reactions.

**Figure 3:**
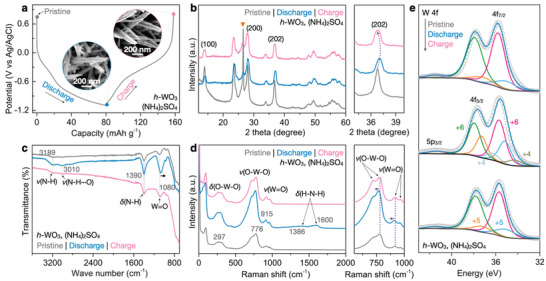
Study on the NH_4_^+^ storage mechanism. a) Charge–discharge profile of *h*‐WO_3_ in (NH_4_)_2_SO_4_ at 1 A g^–1^. Ex situ b) XRD, c) FTIR, d) Raman, and e) W 4f XPS characterization of the *h*‐WO_3_ electrodes at the selected state of charge. The triangle in b marks the diffraction peak from carbon paper substrate.

As we mentioned earlier, the *h*‐WO_3_ maintains high structural stability because the NH_4_^+^ ions could travel through and be readily accommodated within the ionic tunnels. This is confirmed by the SEM observation that the overall morphology of the *h*‐WO_3_ does not change upon charging and discharging (**Figure** **3**a; Figure S7, Supporting Information). We further conducted ex situ XRD measurement at selected states of charge. The diffraction peaks generally become broader, indicating that the intercalation of NH_4_^+^ ions reduces the crystallinity of *h*‐WO_3_ (Figure 3b). In addition, during discharge (corresponding to the NH_4_^+^ insertion process), the peaks shift to a higher degree, which suggests a contraction of the crystal lattice. This could be because the water molecules within the *h*‐WO_3_ tunnels are (partially) replaced by the NH_4_^+^ ions with smaller ionic sizes (approximately 2.75 vs 1.48 Å). Notably, the structure of *h*‐WO_3_ reversibly expands back upon charging (i.e., NH_4_^+^ desertion). The structural contraction confirms the intercalation of NH_4_^+^ into the *h*‐WO_3_ tunnels and their interactions. The bonding chemistry of NH_4_^+^ ions with *h*‐WO_3_ host was further demonstrated by the Fourier transform infrared (FTIR) spectra. The IR peak at ≈3189 cm^–1^ is ascribed to the N–H stretch of a nonbonded H, whereas the one at ≈3010 cm^–1^ corresponds to the N–H that bonded with the *h*‐WO_3_ host through H···O bonding (Figure 3c).^[^ ^24^ ^]^ We noticed that the pristine sample also shows the former peak, which could be due to the preintercalated NH_4_^+^ that was introduced during the synthesis process. The latter peak appears upon discharging and then becomes weakened during charging, which suggests the building and breaking of hydrogen bonds between the NH_4_^+^ and *h*‐WO_3_ host. In addition, the peak at ≈1080 cm^–1^ that is ascribed to the W═O bond stretches shifts to lower frequency,^[^ ^25^ ^]^ which is caused by the reversible reduction of W^6+^ to W^5+^/W^4+^ during the insertion of NH_4_^+^ into the *h*‐WO_3_ framework. The peak intensity and position recover back upon charging and are nearly identical to the initial state, which demonstrates that the insertion and desertion of NH_4_^+^ ions are highly reversible. This result was further supported by the Raman analysis (Figure 3d). The characteristic peaks of NH_4_^+^ are observed upon discharging. As a result of NH_4_^+^ insertion, the W^6+^ is partially reduced to W^5+^/W^4+^. The bonds of W^5+/4+^—O and W^5+/4+^═O are weaker than those involving W^6+^ ions due to the lower ionic charge, therefore resulting in a redshift of these peaks.^[^ ^26^ ^]^ The reduction of W^6+^ to W^5+^/^4+^ during NH_4_^+^ insertion was also revealed by the X‐ray photoelectron spectroscopy (XPS) analysis (Figure 3e). The pristine *h*‐WO_3_ is composed of both the W^6+^ and W^5+^ species, suggesting that *h*‐WO_3_ may contain oxygen vacancies. After the discharge process, W^4+^ can be identified on the *h*‐WO_3_ surface, indicating the insertion of NH_4_^+^ that causes the reduction of high valent W species. Whereas after charging, the surface oxidation state of W reverses back, again confirming that the NH_4_^+^ insertion/desertion is highly reversible and does not induce a significant structural change of the *h*‐WO_3_ framework. These analyses suggest that the NH_4_^+^ ions can reversibly intercalate into and interact with the *h*‐WO_3_ through the hydrogen bonding chemistry, which enables ultrafast kinetics.

**Figure 4:**
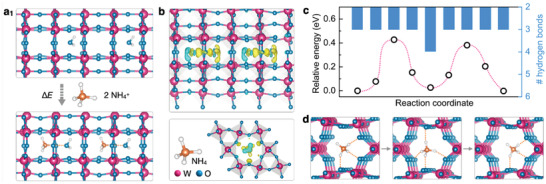
The simulated intercalation process of NH_4_^+^ ions into *h*‐WO_3_ tunnels. a) The lowest‐energy configuration of *h*‐WO_3_ with intercalated NH_4_^+^ ions. b) The charge density difference shows the movement of charge due to the interaction between NH_4_^+^ and *h*‐WO_3_. c) The migration path of NH_4_^+^ ions along the *h*‐WO_3_ tunnels and the corresponding migration energy barriers. d) The initial state, transition state, and final state of NH_4_^+^ ions migration.

The NH_4_^+^ insertion process was further simulated using the density functional theory (DFT) calculations. The lower‐energy model structure of NH_4_^+^ inserted *h*‐WO_3_ was first constructed. The energy variation caused by the insertion of one NH_4_^+^ ion was −11.32 eV (Table S1, Supporting Information), and the host–guest interaction energy was 2.83 eV for NH_4_ per bonding, which suggests that the insertion of NH_4_^+^ ions would result in distortions of the *h*‐WO_3_ host. Indeed, the interaction of NH_4_^+^ and *h*‐WO_3_ was found to result in an increase in the W═O bond length from 2.033 to 2.058 Å, which explains the redshifts of the W = O peaks in FTIR and Raman spectra. We also found that the inserted NH_4_^+^ interacts with not only the *h*‐WO_3_ but also the neighboring H_2_O through the hydrogen bonding (**Figure** **4**a). The building of hydrogen bonds between the NH_4_^+^ ions and *h*‐WO_3_ provides a channel for the electron transfer (2.88 electrons in total) from the oxide to NH_4_^+^ as demonstrated by the Bader charge analysis (Figure 4c; Table S2, Supporting Information). As a result, the charge localization is accumulated mostly on the terminal oxygen atoms that are coordinated with NH_4_^+^ ions, which benefits the fast charge transfer.^[^ ^27^ ^]^ We then simulated the transport of NH_4_^+^ ions along the ionically conductive tunnels within *h*‐WO_3_. The NH_4_^+^ initially forms three hydrogen bonds with the *h*‐WO_3_ with an intercalation energy barrier of 0.42 eV (Figure 4b) and then diffuses further into the channel, forming four hydrogen bonds (Figure 4d). Unlike metallic cations such as Li^+^ that are spherical, the NH_4_^+^ has the shape of a regular tetrahedron with four hydrogen atoms located at the vertices at equal distances from the nitrogen center. This configuration allows the NH_4_^+^ to twist and rotate during the diffusion to the *h*‐WO_3_ tunnels. The movement is similar to swinging on monkey bars. As the NH_4_^+^ migrates, one of the hydrogen atoms disconnects with the oxygen of the *h*‐WO_3_ by breaking the hydrogen bonds, and then moves forward to build a new hydrogen bond with the oxygen atom in the front. In contrast, the other three hydrogen atoms remain connected. The breaking and building of hydrogen bonds take place continuously and alternately, which ensures a smooth diffusion of NH_4_^+^ into the *h*‐WO_3_ host and might account for the good rate capability of NH_4_^+^.

We further fabricate an NH_4_^+^ full cell with *h*‐WO_3_ anode and (NH_4_)_0.5_V_2_O_5_ cathode. (NH_4_)_0.5_V_2_O_5_ possesses a nanobelt morphology (Figure S9, Supporting Information) with super rate capacity (see Figure S9a,b in the Supporting Information; 57.6 mAh g^–1^ at 1 A g^–1^; 51.3 mAh g^–1^ at 10 A g^–1^) and cycling stability (see Figure S9c in the Supporting Information; ≈100% capacity retention after 5000 cycles at 5 A g^–1^). Given the electrochemical performance of (NH_4_)_0.5_V_2_O_5_ nanobelts, the optimized active mass ratio between cathode and anode should be 1:1. As shown in Figure S9d (Supporting Information), the full cell provides a reversible capacity of about 40 mAh g^–1^ at 1 A g^–1^ between 0 and 1.85 V based on the active mass of both electrodes (similarly from now on). The energy density and power density are up to about 34 Wh kg^–1^ and 4300 W kg^–1^ (Figure S8e, Supporting Information). Furthermore, the (NH_4_)_0.5_V_2_O_5_//*h*‐WO_3_ full cell delivers ultralong cycling stability over 50 000 cycles (capacity retention is about 71%).

In summary, we suggest that the mixed ionic–electronic conductors are promising electrode materials for NH_4_^+^ ion batteries. Specifically, we proposed *h*‐WO_3_ with a hexagonal tunnel structure as a suitable host for NH_4_^+^ storage. Thanks to the mixed ionic–electronic conductivity, the structural stability, and the rich redox reactions, the *h*‐WO_3_ exhibits high NH_4_^+^ storage of 82 mAh g^–1^ at 1 A g^–1^ along with outstanding cyclic performance up to 200 000 cycles, which are much superior to its polymorph *m*‐WO_3_ that does not present such ionically conductive tunnels. This work not only establishes the *h*‐WO_3_ as a promising electrode material for NH_4_^+^ ion batteries but also provides insights into the role of mixed ionic–electronic conductivity in enhancing the electrochemical performance of rechargeable batteries that use nonmetallic ions as the charge carriers.

## 2 Experimental Section

### Synthesis of *h*‐WO_3_ and *m*‐WO_3_ Nanowires

The *h*‐WO_3_ nanowires were grown on carbon paper substrate via a hydrothermal reaction.^[^ ^28^ ^]^ First, 3.8 mmol Na_2_WO_4_⋅2H_2_O and 11 mmol H_2_C_2_O_4_ were dissolved in 32 and 48 mL of H_2_O to form clear solutions, respectively. The pH of the former was adjusted to ≈1 by adding HCl drops before the two solutions were mixed. After that, 15 mmol (NH_4_)_2_SO_4_ was added to the above mixture, which was then transferred to a 100 mL autoclave and heated at 180 °C for 16 h. The carbon paper with *h*‐WO_3_ nanowires was collected and washed after the reaction. The *m*‐WO_3_ nanowires were obtained by annealing the *h*‐WO_3_ nanowires precursor at 500 °C in the air for 3 h. On average, 3.9 mg cm^–2^ of WO_3_ nanowires is grown on carbon paper substrate.

### Synthesis of (NH_4_)_0.5_V_2_O_5_ Nanobelts

The (NH_4_)_0.5_V_2_O_5_ nanobelts were synthesized by one‐step hydrothermal method according to previous work.^[^ ^29^ ^]^ Typically, NH_4_VO_3_ (0.29 mg) was dissolved in 35 mL deionized water at 60 ℃. Then, *β*‐cyclodextrin (0.34 mg) was added. After continuously stirred for 1 h, H_2_C_2_O_4_ (0.34 mg) was added to the above solution to form a bottle green solution. The solution was then transferred into a 50 mL Teflon‐lined autoclave at 120 ℃ for 10 h. After natural cooling, the products were filtered and washed with deionized water and ethyl alcohol, and then dried at 80 °C for 24 h under vacuum.

### Material Characterization

The products were characterized by X‐ray diffraction (XRD, Bruker D8 Advance), scanning electron microscopy (SEM, FEI Nova Nano 630), transmission electron microscopy (TEM, FEI Titan CM30), Fourier‐transform infrared spectroscopy (FTIR, Nicolet iS10), X‐ray photoelectron spectroscopy (XPS, Amicus/ESCA 3400), Raman spectroscopy (LabRAM HR), and TG/DSC (TA STD650).

### Electrochemical Measurements

The electrochemical measurements were conducted in a three‐electrode cell, where the *h*‐WO_3_ (or *m*‐WO_3_) on carbon paper was used as the working electrode, a Ag/AgCl electrode, and a graphite rod were used as the reference electrode and counter electrode, respectively. The electrolyte was either 1 m (NH_4_)_2_SO_4_ or 1 m LiClO_4_.

### Simulations

The present first principle DFT calculations are performed by Vienna Ab initio Simulation Package (VASP)^[^ ^30^ ^]^ with the projector augmented wave (PAW) method.^[^ ^31^ ^]^ The exchange‐functional is treated using the generalized gradient approximation (GGA) of Perdew–Burke–Ernzerhof (PBE) functional.^[^ ^32^ ^]^ The energy cutoff for the plane wave basis expansion was set to 450 eV and the force on each atom less than 0.01 eV Å^−1^ was set for the convergence criterion of geometry relaxation. A 1 × 1 × 4 supercell of WO_3_ was adopted for further simulation. The Brillouin zone integration is performed using 2 × 2 × 1 *k*‐point sampling. The self‐consistent calculations apply a convergence energy threshold of 10^–5^ eV. The DFT‐D3 method was employed to consider the van der Waals interaction.^[^ ^33^ ^]^ Transition state searching was calculated using the climbing‐image nudged elastic band (CI‐NEB) method.^[^ ^34^ ^]^ The adsorption energy of NH_4_^+^ was calculated according to *E*_ads_ = (*E*_hybrid_ − *E*_initial_ − 2 *E*_ammonium_)/2, where *E*_hybrid_ is the total energy of the NH_4_^+^ adsorbed systems, and *E*_initial_ and *E*_ammonium_ are the energies of the initial WO_3_ structure and the NH_4_^+^, respectively.

## Conflict of Interest

The authors declare no conflict of interest.

## Supporting information

Supporting Information

## Data Availability

The data that support the findings of this study are available from the corresponding author upon reasonable request.
