# Supplementary material for: Ionically Conductive Tunnels in h‐WO3 Enable High‐Rate NH4 + Storage
Source: Adv Sci (Weinh). 2022 Feb 2;9(10):2105158. doi: 10.1002/advs.202105158 (PMC8981906; doi:10.1002/advs.202105158)
Supplement: Supplementary file 1 — Supporting Information [file ADVS-9-2105158-s001.pdf]

ADVANCED SCIENCE
Open Access

Supporting Information

for *Adv. Sci.*, DOI 10.1002/advs.202105158

Ionically Conductive Tunnels in *h*-WO_3 Enable High-Rate NH_4^+ Storage

Yi-Zhou Zhang, Jin Liang, Zihao Huang, Qian Wang, Guoyin Zhu, Shengyang Dong, Hanfeng Liang* and Xiaochen Dong**

WILEY-VCH

ADVANCED SCIENCE
Open Access

Supporting Information

for *Adv. Sci.*, DOI: 10.1002/advs.202105158

Ionically Conductive Tunnels in h-WO3 Enable High Rate NH4+ Storage

Yi-Zhou Zhang, Jin Liang, Zihao Huang, Qian Wang, Guoyin Zhu, Shengyang Dong, Hanfeng Liang,* Xiaochen Dong**

Supporting Information

Ionically Conductive Tunnels in h-WO_3 Enable High Rate NH_4^+ Storage

Yi-Zhou Zhang, Jin Liang, Zihao Huang, Qian Wang, Guoyin Zhu, Shengyang Dong, Hanfeng Liang,* Xiaochen Dong**

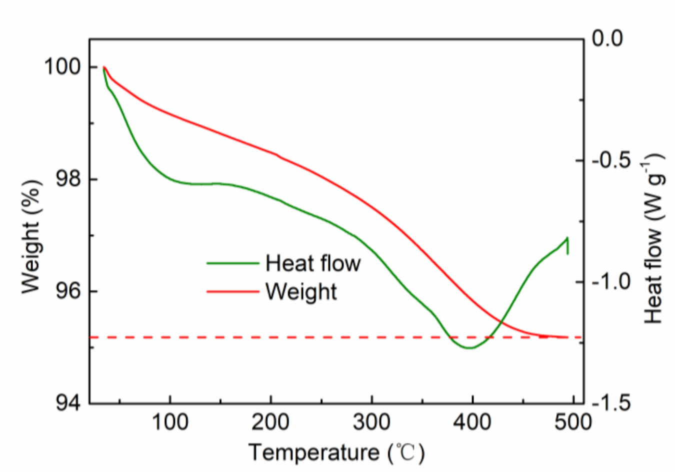

Figure S1. TG/DSC profile of *h*-WO_3 powder.

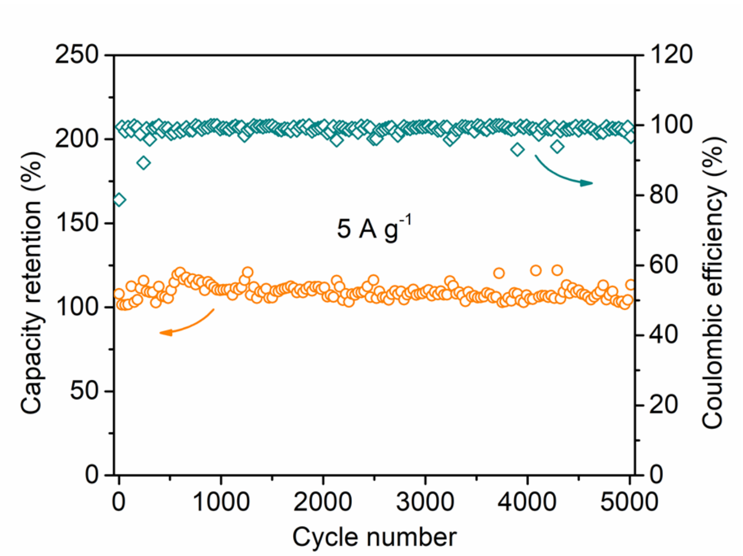

Figure S2. Cycling performance of *h*-WO_3 in 1 M $(NH_4)_2SO_4$ at 5 A g^{-1}.

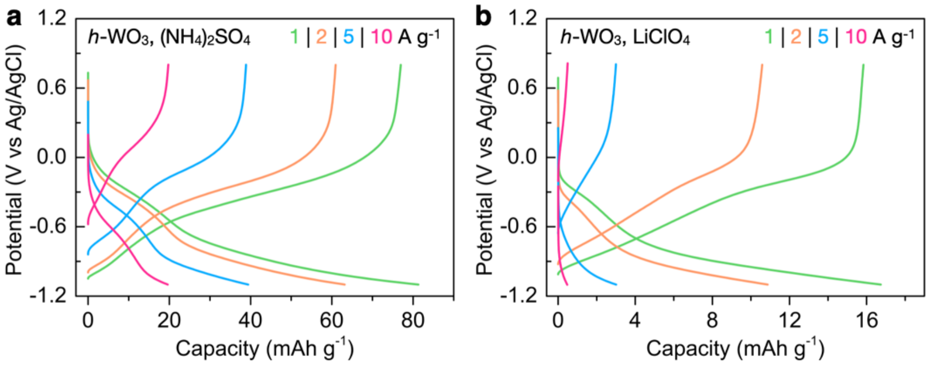

Figure S3. The charge-discharge curves of *h*-WO_3 electrode with 1 M $(NH_4)_2SO_4$ (a) and $LiClO_4$ (b) as the electrolyte under various current densities.

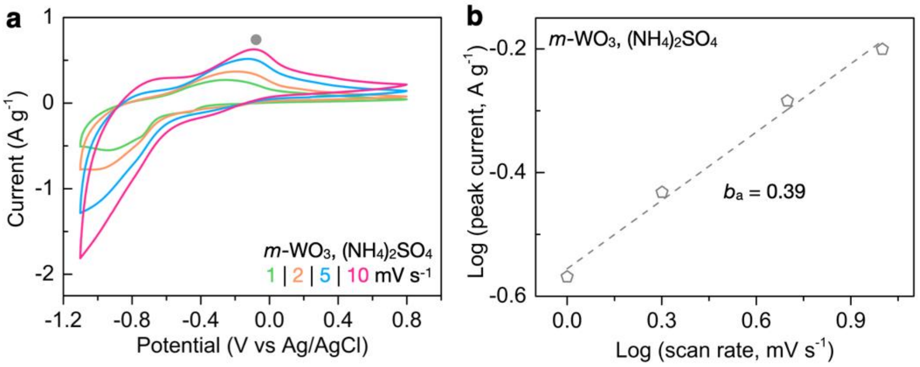

Figure S4. (a) CV curves at different scan rates and (b) the *b* value of *m*-WO_3 in 1 M $(NH_4)_2SO_4$ electrolyte.

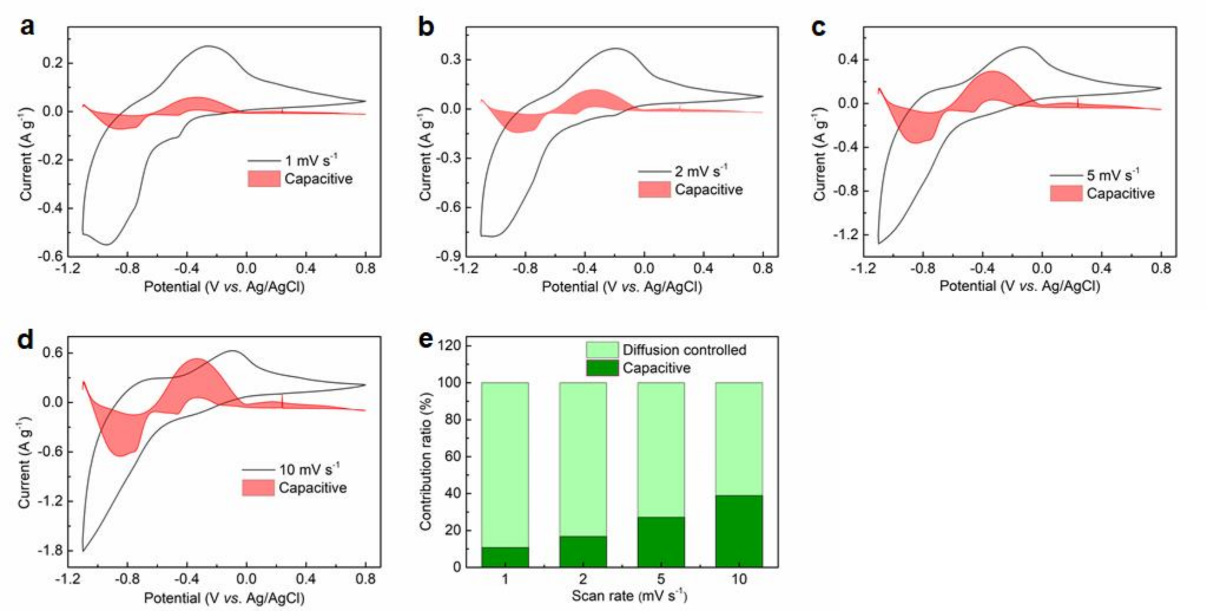

Figure S5. Separation of the capacitive and diffusion currents of *m*-WO_3 at (a) 1 mV s^{-1}, (b) 2 mV s^{-1}, (c) 5 mV s^{-1}, (d) 10 mV s^{-1}. (e) Contribution of the capacitive behavior of 1 (10.7%), 2 (16.7%), 5 (21.1%) and 10 (38.8%) mV s^{-1}.

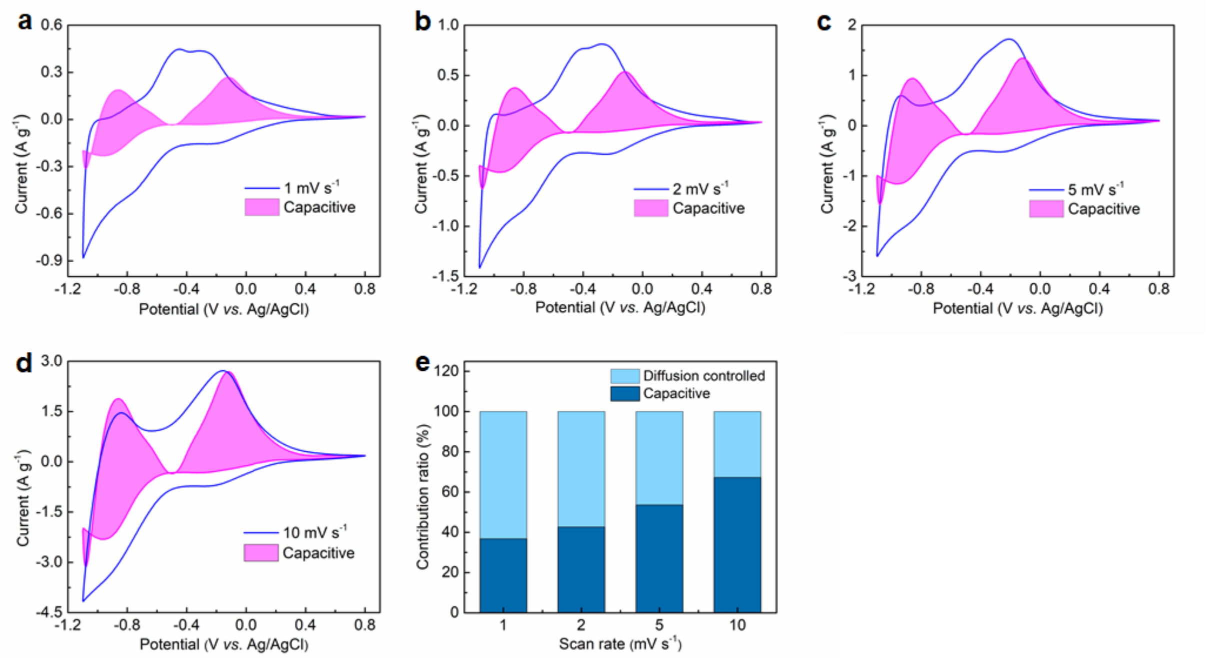

Figure S6. Separation of the capacitive and diffusion currents of *h*-WO_3 at (a) 1 mV s^{-1}, (b) 2 mV s^{-1}, (c) 5 mV s^{-1}, (d) 10 mV s^{-1}. (e) Contribution of the capacitive behavior of 1 (36.8%), 2 (42.7%), 5 (53.6%) and 10 (67.3%) mV s^{-1}.

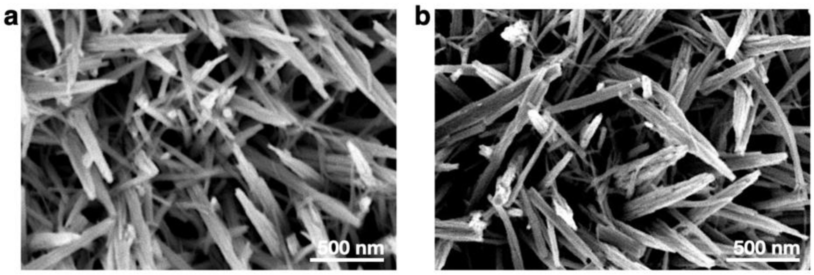

Figure S7. SEM images of the *h*-WO_3 after (a) discharge and (b) charge.

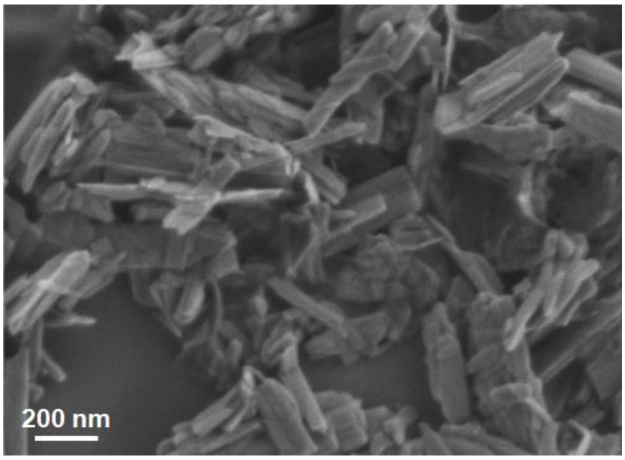

Figure S8. SEM image of $(NH_4)_{0.5}V_2O_5$.

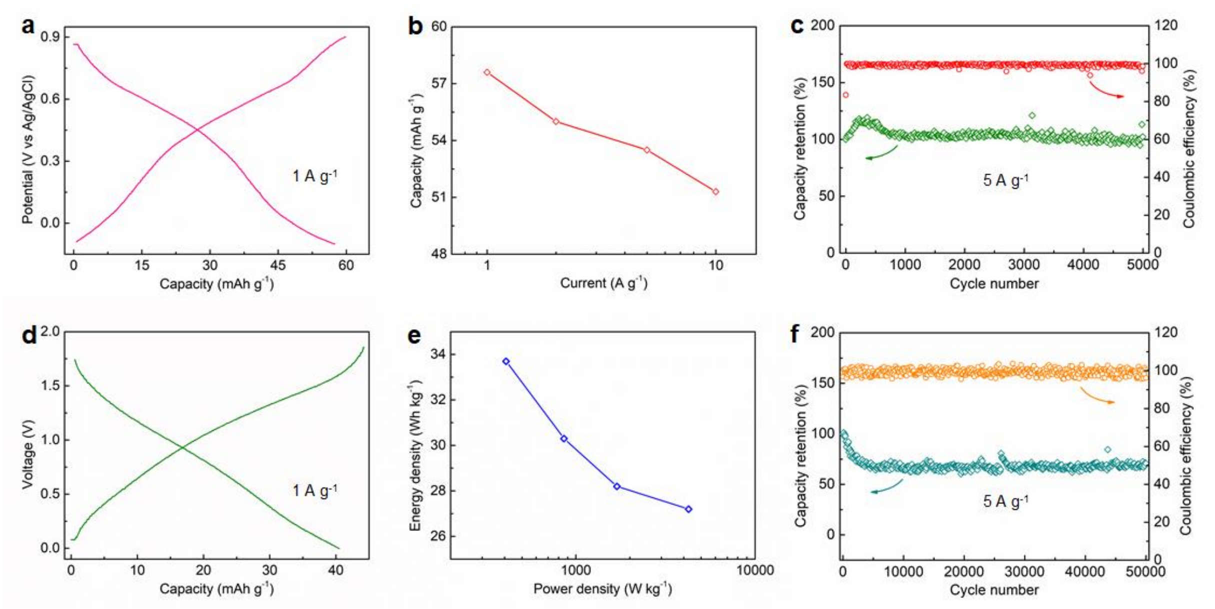

Figure S9. Electrochemical performance of $(NH_4)_{0.5}V_2O_5$ nanobelts. (a) Typical CD curve at a current density of 1 A g^{-1}. (b) Rate capacities. (c) Cycling stability at 5 A g^{-1}. Electrochemical performance of $(NH_4)_{0.5}V_2O_5$//h-WO_3 full cell. (d) Typical CD curve at a current density of 1 A g^{-1}. (e) Ragone curves. (f) Cycling stability at 5 A g^{-1}.

Table S1. Calculated energies (eV) for the *h*-WO_3 and NH_4^+ intercalated *h*-WO_3.

$\boldsymbol{E}_{\mathbf{initial}}$	$\boldsymbol{E}_{\mathbf{ammonium}}$	$\boldsymbol{E}_{\mathbf{hybrid}}$	$\Delta\boldsymbol{E}$
-472.60	-17.61	-530.45	-11.32

The values listed above (from left to right) present the energies of the initial state (pristine *h*-WO_3 without NH_4^+ intercalation), one NH_4^+ ion, and hybrid state (*h*-WO_3 with two intercalated NH_4^+ ions), respectively. The variation of intercalation energy caused by one NH_4^+ insertion (ΔE) was calculated according to $\Delta E = [E_{\text{hybrid}} - (2\ E_{\text{ammonium}} + E_{\text{initial}})]/2$, whereas the average guest-host interaction energy for NH_4 (4 bonds) per bonding was $\Delta E/4$.

Table S2. The charge and charge transfer of two NH_4^+ ions upon insertion into *h*-WO_3.

**Atom**	**Charge**	**Charge transfer**
N	6.299876	-0.700124
H	0.461923	-0.538077
H	0.429611	-0.570389
H	0.454021	-0.545979
H	0.478277	-0.521723
N	6.300366	-0.699634
H	0.461724	-0.538276
H	0.429622	-0.570378
H	0.453892	-0.546108
H	0.478061	-0.521939
Per NH_4^+		-2.876314
